# Supplementary material for: Mechanisms governing the pioneering and redistribution capabilities of the non-classical pioneer PU.1
Source: Nat Commun. 2020 Jan 21;11:402. doi: 10.1038/s41467-019-13960-2 (PMC6972792; doi:10.1038/s41467-019-13960-2)
Supplement: Supplementary file 7 — Source data [file 41467_2019_13960_MOESM7_ESM.zip › Source_Data/FigureS2/FigureS2B_MotifScanOutput/KmeansCluster7/homerResults/motif26.similar.html]

motif26

## Information for motif26

A
G
T
C
G
T
C
A
C
G
T
A
A
G
T
C
A
G
T
C
C
G
T
A
A
C
T
G
C
G
T
A
A
C
G
T
A
G
T
C
A
C
G
T
  
Reverse Opposite:  

C
G
T
A
A
C
T
G
C
G
T
A
A
C
G
T
A
G
T
C
A
C
G
T
A
C
T
G
C
T
A
G
A
C
G
T
A
C
G
T
A
C
T
G
  

|  |  |
| --- | --- |
| p-value: | 1e-49 |
| log p-value: | -1.150e+02 |
| Information Content per bp: | 1.938 |
| Number of Target Sequences with motif | 125.0 |
| Percentage of Target Sequences with motif | 3.89% |
| Number of Background Sequences with motif | 263.0 |
| Percentage of Background Sequences with motif | 0.60% |
| Average Position of motif in Targets | 95.9 +/- 35.0bp |
| Average Position of motif in Background | 97.1 +/- 67.7bp |
| Strand Bias (log2 ratio + to - strand density) | 0.0 |
| Multiplicity (# of sites on avg that occur together) | 1.00 |
| Motif File: | file (matrix) reverse opposite |

### Similar de novo motifs found

|  |  |  |  |  |  |  |  |
| --- | --- | --- | --- | --- | --- | --- | --- |
| Rank | Match Score | Redundant Motif | P-value | log P-value | % of Targets | % of Background | Motif file |
| 1 | 0.888 | C G T A A T C G G T C A A C G T G T A C A C G T A T C G T C A G A C G T | 1e-31 | -72.550169 | 3.36% | 0.75% | motif file (matrix) |
| 2 | 0.800 | C G T A A G T C A T G C G T C A A T C G C G T A A C G T | 1e-26 | -60.966994 | 13.52% | 7.72% | motif file (matrix) |
